# Correlated chromosomal periodicities according to the growth rate and gene expression

**DOI:** 10.1038/s41598-020-72389-6

**Published:** 2020-09-23

**Authors:** Liu Liu, Masaomi Kurokawa, Motoki Nagai, Shigeto Seno, Bei-Wen Ying

**Affiliations:** 1 Graduate School of Life and Environmental Sciences, University of Tsukuba, 1-1-1 Tennodai, Tsukuba, Ibaraki 305-8572 Japan; 2 Graduate School of Information Science and Technology, Osaka University, 1-5 Yamadaoka, Suita, Osaka 565-0871 Japan

**Keywords:** Computational biology and bioinformatics, Microbiology, Systems biology

## Abstract

Linking genetic information to population fitness is crucial to understanding living organisms. Despite the abundant knowledge of the genetic contribution to growth, the overall patterns/features connecting genes, their expression, and growth remain unclear. To reveal the quantitative and direct connections, systematic growth assays of single-gene knockout *Escherichia coli* strains under both rich and poor nutritional conditions were performed; subsequently, the resultant growth rates were associated with the original expression levels of the knockout genes in the parental genome. Comparative analysis of growth and the transcriptome identified not only the nutritionally differentiated fitness cost genes but also a significant correlation between the growth rates of the single-gene knockout strains and the original expression levels of these knockout genes in the parental strain, regardless of the nutritional variation. In addition, the coordinated chromosomal periodicities of the wild-type transcriptome and the growth rates of the strains lacking the corresponding genes were observed. The common six-period periodicity was somehow attributed to the essential genes, although the underlying mechanism remains to be addressed. The correlated chromosomal periodicities associated with the gene expression-growth dataset were highly valuable for bacterial growth prediction and discovering the working principles governing minimal genetic information.

## Introduction

Connecting genes, their expression and cell growth is a critical issue for understanding living organisms. The growth contributions of genes in terms of their genetic sequences or expression were largely investigated; however, the direct connection and the overall patterns/features of the genes, their expression levels and the growth rates remained unclear.

Genes were often considered definitive factors for bacterial growth. To link genes to growth, experimental growth assays of genetically differentiated *Escherichia coli* (*E. coli*) strains were performed to a large extent. The genetic contribution to growth was first evaluated determinatively; that is, genes were identified as either essential or nonessential for cell survival^1,2^. Further detailed growth profiles related to the individual nonessential genes were determined systematically^3,4^, based on a landmark study involving the construction of single-gene knockout strains, i.e., the Keio collection^2^. The high-throughput and multilevel analyses of the Keio collection not only provided the morphological profiles related to the individual genes^5–7^ but also determined the conditional essentiality of these nonessential genes^8,9^. Moreover, analysing the contribution of the genomic fragments, rather than a single gene, to bacterial growth was applied as an alternative approach. Successful genome reduction^10–12^ resulted in the common finding that the fitness decrease was attributed to the deletion of genomic fragments^13–15^. In particular, a quantitative relationship between genome reduction and growth was detected; that is, the growth rates of the genome-reduced strains declined in a deletion length-dependent manner^13^.

In addition, bacterial growth is known to be associated with gene expression^16^. In addition to the static snapshots of transcriptomes, highlighting the global views of the gene networks^17–19^, growth-coordinated gene expression was identified by observing the genetically identical strain under varied growth conditions^20–22^. Intriguing findings were often reported, as the genes could be statistically categorized into clusters in a growth-dependent or stress-responsive manner^23,24^, although they were generally regulated in their own specific way in response to environmental changes^25,26^. Our previous study of a genome-reduced strain showed that the expression levels of the genes were either positively or negatively correlated with growth rates^21^. These results indicated that the expression levels of the individual genes were quantitatively associated with the fitness of the growing bacterial population.

Taken together, studies on the quantitative contribution of genetic information to growth fitness have mainly focused on two aspects: growth assays of genetically deficient strains and transcriptome analysis of regular strains. As the two types of studies were usually performed independently, the global features/patterns directly connecting the gene, the expression and the growth remained uncertain. To determine the direct linkage between the growth contribution of the individual genes and their original expression levels in the wild-type genome/strain, we analysed the growth rates of the single-gene knockout *E. coli* strains (Keio collection) in parallel and the original expression levels of these genes in the parental strain used for constructing the Keio collection. Comparative analyses of the growth rates and the transcriptomes were performed to discover the direct linkages among the genes, their expression and cell growth as well as the overall patterns/features of the linkages.

## Results

### Linkage of the non-essential genes to the growth rates

**Figure 1:**
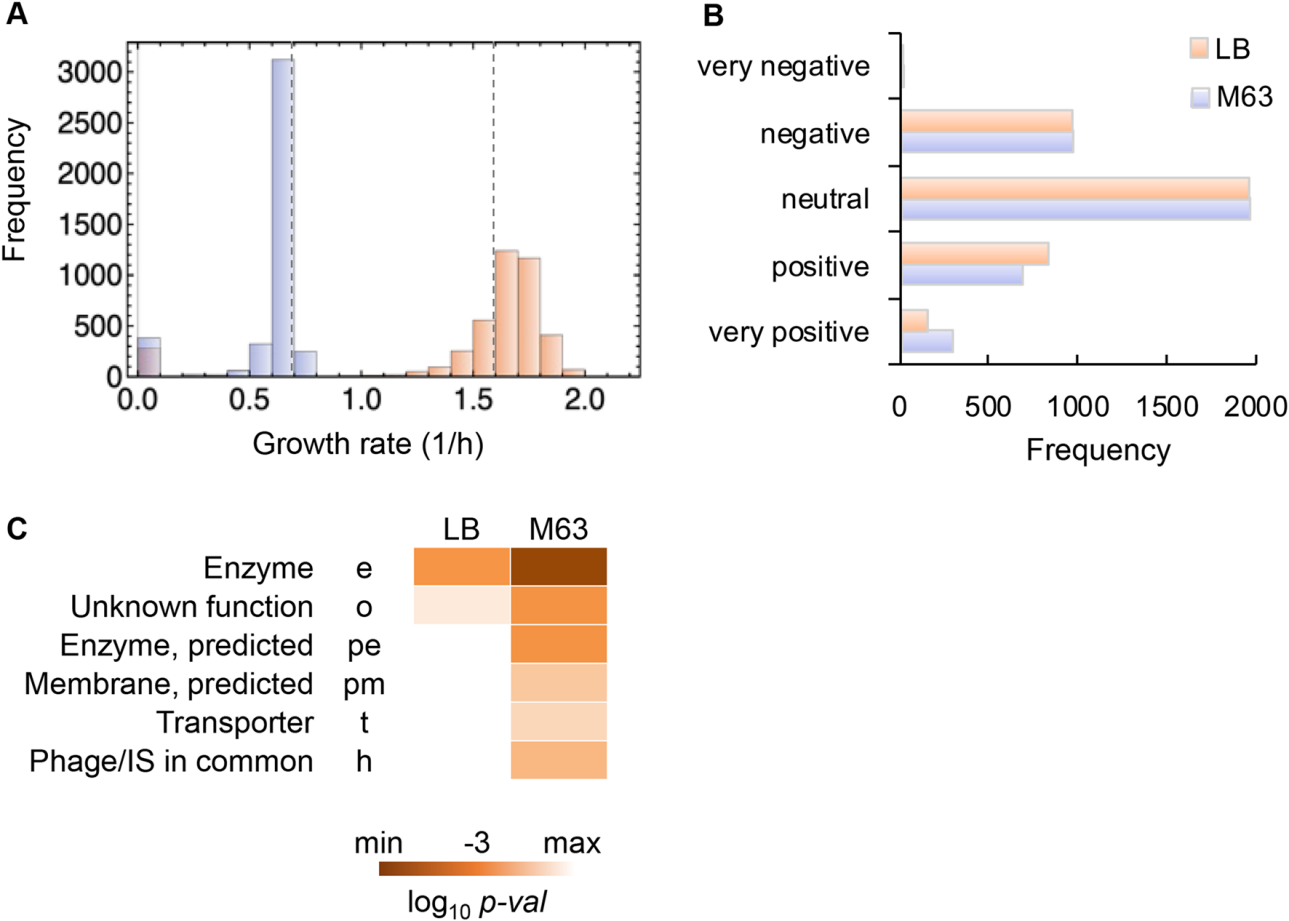
Growth rates of the single-gene knockout strains. **(A)** Distributions of the growth rates. Exponential growth rates of the single-gene knockout strains (N = 3,909) grown in LB and M63 media are shown in peach and lilac, respectively. The growth rates of the wild-type strain BW25113 in both media are indicated with broken lines. (**B)** Gene classification based on the growth rate. According to the box-and-whisker plot (Fig. S1), five gene classes were newly defined in the growth conditions in LB and M63 media, which are shown in peach and lilac, respectively. Very negative, negative, neutral, positive, and very positive indicate the growth rates of the single-gene knockout strains within the ranges of the outliers greater than the 1.5 interquartile range (IQR), from the 1.5 IQR to the upper quartile, from the upper to the lower quartiles, from the lower quartile to the 1.5 IQR, and within the ranges of outliers lower than 1.5 IQR, respectively. (**C)** Heatmap of the enriched gene categories for the very positive class. The gradation from light to dark orange indicates the statistical significance of the enrichment analysis from low to high, respectively.

The non-essential genes were categorized according to the growth rates of the single-gene knockout strains. A novel classification of gene function was performed according to the exponential growth rate of the *E. coli* strain lacking the corresponding gene. Both the single-gene knockout strains and their parental strain, the wild-type strain BW25113, were subjected to the growth assay in LB and M63 media. The results showed that the absence of a single nonessential gene triggered either an increase or a decrease in the growth rate, although the absence of most genes insignificantly disturbed growth (Fig. 1A). In comparison to the growth rate of the parental strain, the growth rates of a large number of the single-gene knockout strains were reduced in the minimal medium M63 (Fig. 1A, blue) but increased in the LB medium (Fig. 1A, orange). The growth media-induced differentiation of the distributions of the growth rates intriguingly revealed that most of the non-essential genes were costly under nutrient-rich conditions.

The non-essential genes were statistically categorized into five classes (Fig. 1B) based on the distributions of the growth rates (Fig. S1). If the gene positively contributed to the growth in the wild-type (parent) strain, the absence of this gene would lead to a decrease in growth in the corresponding knockout strain. The high and low growth rates of the single-gene knockout strains represented the negative and positive contributions of the missing genes to growth. A total of 10 and 14 genes were determined to have very negative effects on growth in LB and M63 media, respectively (Fig. 1B, Table S1). This result indicated that these genes were highly redundant in the wild-type strain. Since no overlapping genes were found for the classes of genes with very negative effects on growth in LB and M63 media (Table S1), genetic redundancy resulted from nutritional differentiation but not nutritional enrichment.

A total of 151 and 293 genes showed very positive contributions to growth in LB and M63 media, respectively (Fig. 1B). The functional enrichment of these genes showed that the genes assigned to the enzyme gene category^27^ (e) were highly significant (*p* < 0.001) for growth in both media (Fig. 1C). In addition, these genes partially overlapped in LB and M63 media (Fig. S2A) and were significantly enriched in enzyme-encoding genes (Fig. S2B). These results indicated the common requirement for the non-essential genes for growth maintenance, regardless of the nutritional conditions. Many more gene categories were enriched in cells in M63 media and largely comprised genes of unknown or predicted function (Fig. 1C, Fig. S2B). This result suggested that genes with unclear function played an important role in *E. coli* grown in poor nutritional conditions. Note that the additional statistical re-evaluation of the mean growth rate of each strain largely reduced the number of the genes divided into the positive and negative classes, nevertheless, the enriched functional gene categories remained the same (Fig. S3).

### Correlation between the growth rates of the knockout strains and the original expression levels of the knockout genes

**Figure 2:**
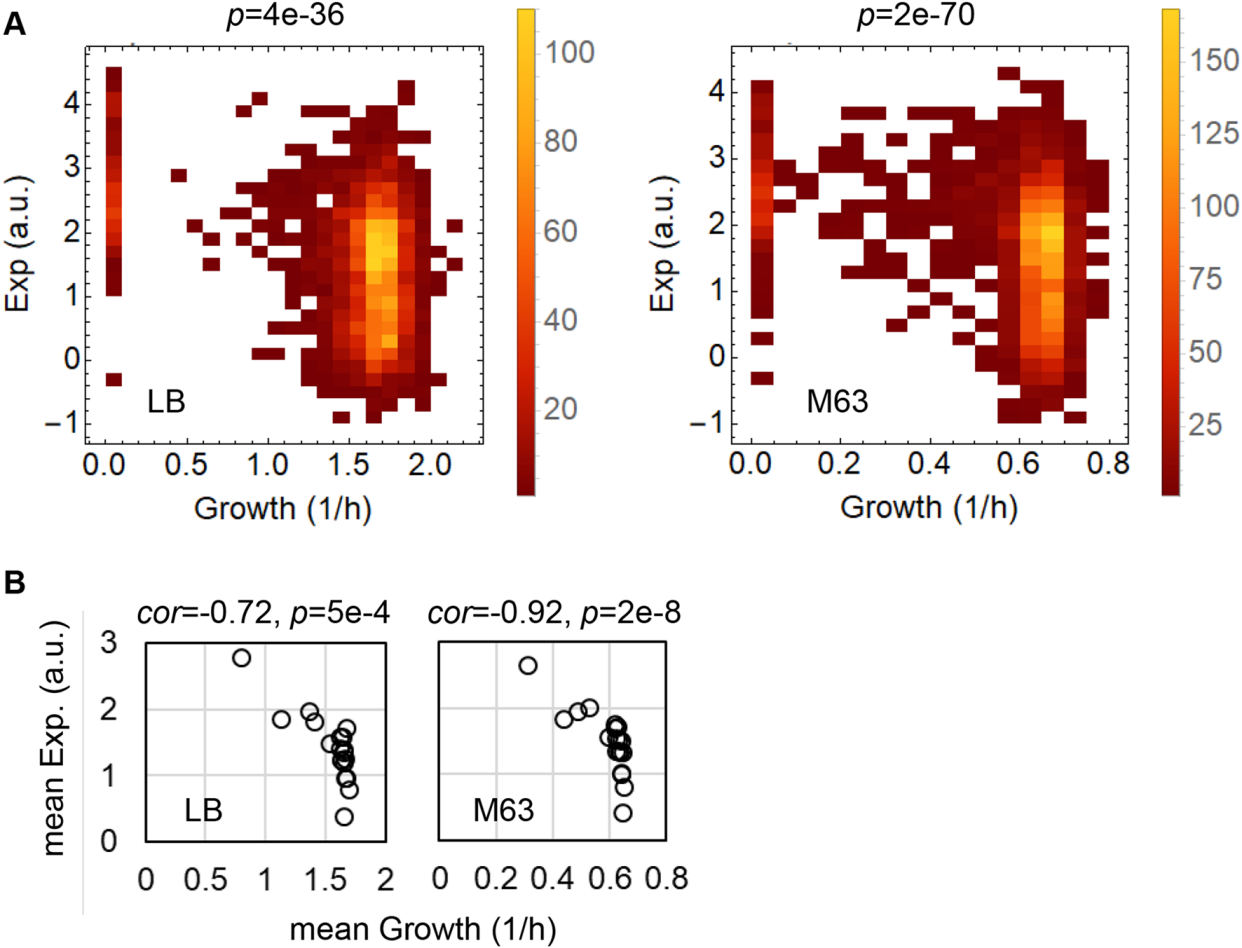
Correlation between growth and expression.** (A)** Density histograms of the growth rates and gene expression. The left and right panels represent the relationships between the expression levels of the genes in the wild-type strain and the growth rates of the knockout strains lacking the corresponding genes in LB and M63 growth conditions, respectively. The colour gradation from red to yellow indicates the number of genes/strains from small to large, respectively. The correlation coefficients are − 0.37 and − 0.39 for LB and M63, respectively. The statistical significance of the Spearman correlation is indicated. (**B)** Scatter plots of the mean growth rates versus the mean expression levels of the gene categories. The open circles represent a total of 19 gene categories, which comprised more than 30 genes. The correlation coefficients and statistical significance are indicated.

The original expression levels of the genes before knockout were determined by transcriptome analysis of the wild-type strain grown in the same media as that used for the growth assay. Weak but statistically significant correlations between the expression levels of the genes in the wild-type strain and the growth rates of the single-gene knockout strains were commonly observed in cells grown in both media (Fig. 2A). It was highly significantly that although the growth rates were determined on the basis of thousands of genetically differentiated strains, they were correlated with the transcriptome of a single strain with a varied genotype. This finding was definitely different from those in previous reports on the coordination of growth with transcriptome reorganization, which generally discussed the relationships between gene expression and the growth rates of the identical strain^20,21,24^. It is unclear if the intriguing correlations were observed by chance; the statistical simulation, in which the data sets on either the growth rates or the gene expression were randomized, was performed 1,000 times each. The results proved that the correlations were statistically significant (Fig. S4).

In addition, the correlations were significant even at the gene category level (Fig. 2B). A total of 19 gene categories containing more than 30 genes were subjected to evaluation (Fig. S5). The expression levels and the growth rates of the genes/strains assigned to the same gene category were averaged. The mean growth rates and the mean expression levels were also correlated in both media (Fig. 2B). Note that the correlations were insignificant within the gene categories (Figs. S6‐S7), except for four categories: enzyme (e), factor (f), structural component (s) and cell process (cp) (*p* < 0.001).

**Figure 3:**
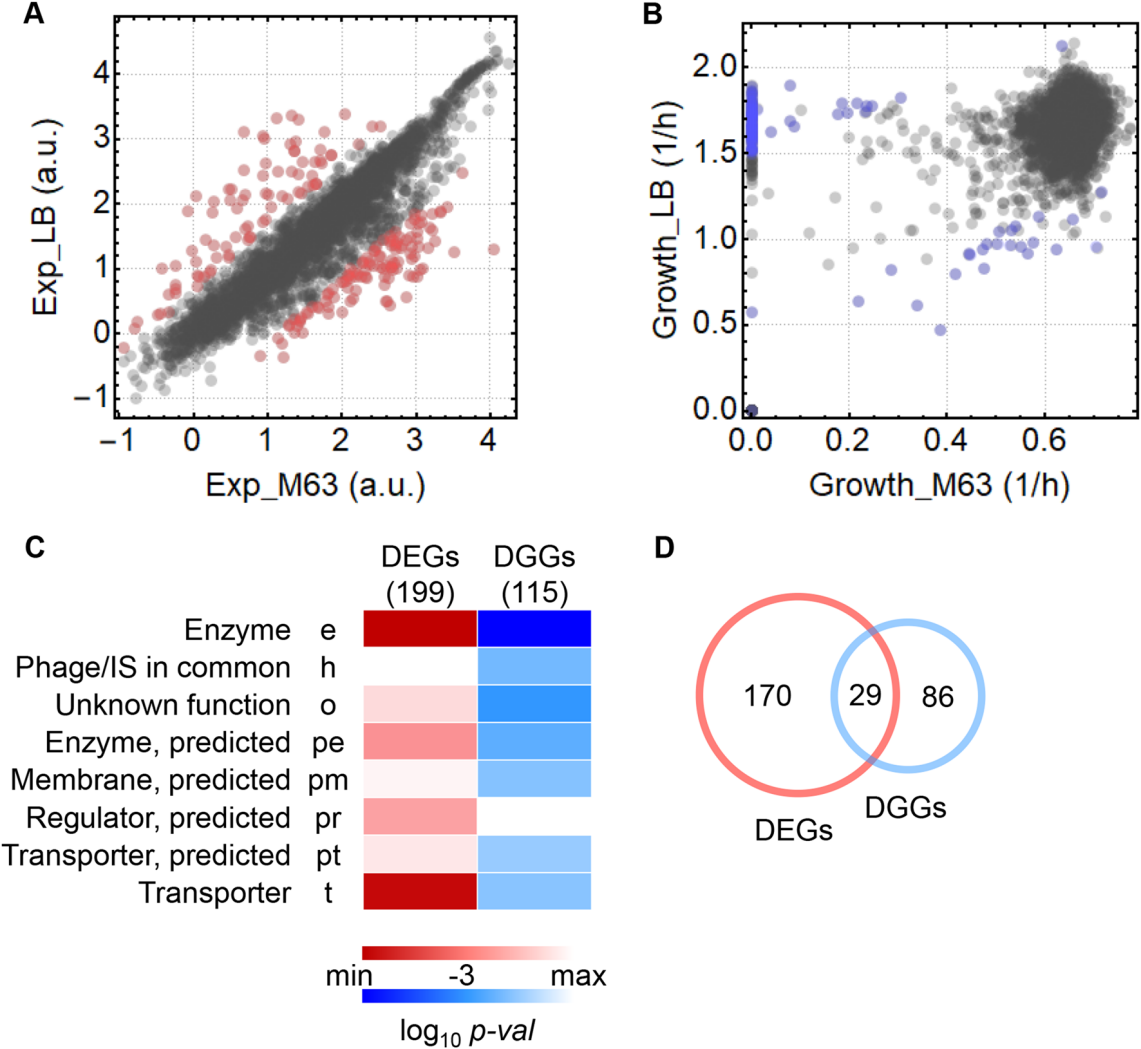
Genes with differential expression and growth in response to media changes.** (A)** Scatter plot of the gene expression of the wild-type strain. Exp_LB and Exp_M63 indicate the expression levels (log_10_RPKM) in LB and M63, respectively. The DEGs in response to media changes are highlighted in red. (**B)** Scatter plot of the growth rates of the single-gene knockout strains. Growth_LB and Growth_M63 indicate the growth rates of the single-gene knockout strains grown in LB and M63, respectively. The strains showing differential growth rates (DGGs) are highlighted in blue. (**C)** Heatmap of the enriched gene categories of DEGs and DGGs. The gradation from light to dark red and blue indicates the statistical significance of the enrichment analysis from low to high for the DEGs and DGGs, respectively. (**D)** Venn diagram of DEGs and DGGs. The numbers of the genes specifically and commonly determined as DEGs and DGGs are indicated.

Moreover, whether the differentially expressed genes (DEGs) corresponded to the strains with differentiated growth rates (DGGs) in response to nutritional changes (*i.e.*, the difference between the two media) was analysed. A total of 199 DEGs (Fig. 3A, red) and 115 DGGs (Fig. 3B, blue) were identified that presented significant fluctuations (FDR *q* < 0.05) in response to media changes. The gene category enrichment analysis showed that the genes assigned to the enzyme (e) and transporter (t) categories and those with undefined functions (o, pe, pm, pr, and pt) were significantly responsive to nutritional changes (Fig. 3C, Fig. S8). In particular, the 29 genes overlapping between the DEGs and DGGs (Fig. 3D) were found to participate in amino acid metabolism and presented the common features of a high growth rate and a low expression level or vice versa (Table S2). This finding not only supported the newly identified correlation between growth and expression (Fig. 2) but also indicated that the genes participating in the biosynthesis of the amino acids arg, cys, leu, met and trp were costly under enriched conditions. Note that the differentiated methods for DEGs determination did not change the enriched gene categories, although the analysis with DESeq2^28^ led to a considerably larger number of DEGs (Fig. S9).

### Common chromosomal periodicities of the growth rates and gene expression

**Figure 4:**
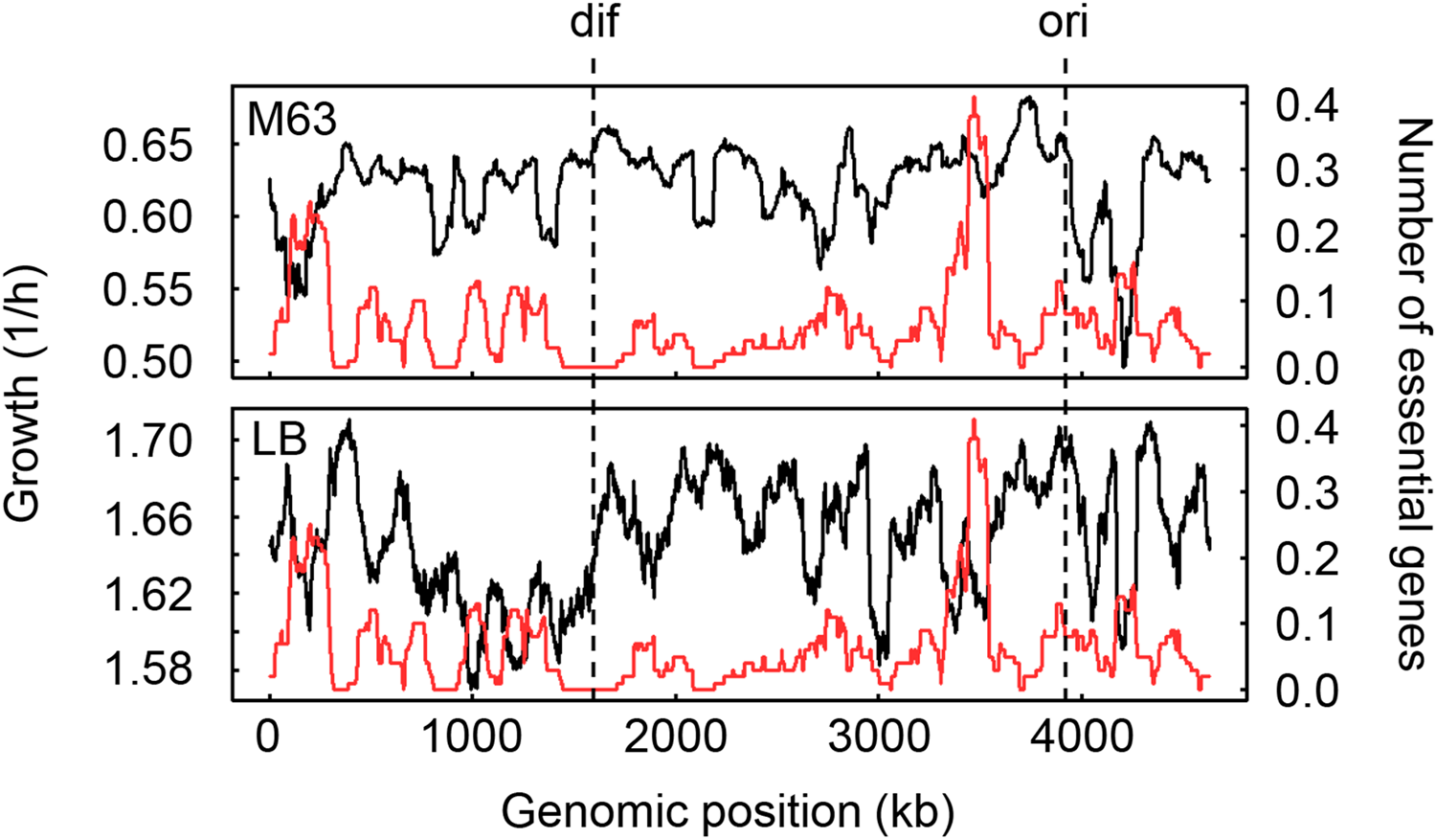
Genomic distributions of the growth rates of the knockout strains. The growth rates of the knockout strains are plotted against the genome of the wild-type genome BW25113 at the genomic positions of the knockout genes, which are the nonessential genes. The mean growth rates of the knockout strains lacking the corresponding genes in every 1kb sliding window are shown in 100-kb bins by the black curves. The genomic distribution of the essential genes is shown by the red curves. The mean numbers of essential genes in every 1kb sliding window are shown in 100-kb bins. The *ori* and *dif* are indicated with broken lines. The upper and bottom panels represent the growth in M63 and LB media, respectively. The Pearson correlation coefficients between the mean growth rates and the mean numbers of essential genes were -0.03 (*p* = 0.06) and − 0.15 (*p* = 3e − 24) in M63 and LB, respectively.

To understand the correlation between the growth rates and gene expression, the genomic distribution of the genes was investigated. No specific pattern was observed by directly plotting the growth rates with respect to the genes at various genomic positions (Fig. S10); thus, the mean growth rates of the strains lacking the genes located within every 100-kb span were calculated and plotted within a 1kb sliding window (Fig. 4, black curves). A significant decrease in the growth rates was observed for cells with deletions close to the *ori* in both media and in the right arm of the genome between the *ori* to *dif* in LB. This result suggested that the genes either close to the *ori* or that were replicated in a clockwise manner governed growth to a great extent, although none of these genes were essential for survival. It is of note that the growth decrease based on the genomic position was not simply a result of the genomic locations of the essential genes, which were excluded from the growth assay. The genomic distribution of the essential genes was independent of the genomic fluctuation of the growth rates (Fig. 4, red curves).

**Figure 5:**
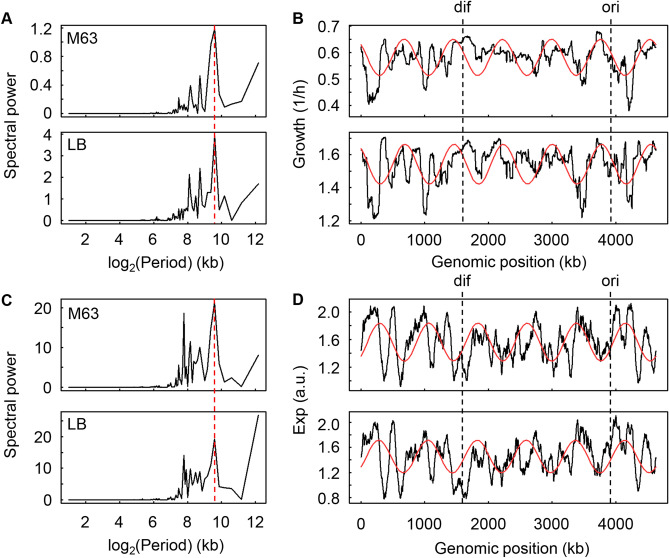
Chromosomal periodicities of the growth rate and gene expression.** (A)** Spectral power of the Fourier-transformed growth rates. The major spectral power at a wavelength of 772 kb is indicated by the broken line in red. **(B)** Periodograms of the growth rates. The growth rates of the strains lacking either the non-essential genes (knockout strains) or the essential genes (zero growth) are plotted against the BW25113 genome. The mean growth rates of the strains lacking the corresponding genes (including essential and nonessential genes) in every 1-kb sliding window are shown in 100-kb bins by black lines. The periodicities of the highest spectral power (**A**, red lines) are shown in the curves in red. The upper and bottom panels indicate growth in M63 and LB, respectively. The Fisher’s g test results for the periods in M63 and LB were 0.168 (*p* = 2e − 182) and 0.146 (*p* = 1e − 155), respectively. The *ori* and *dif* are indicated by broken lines in black. (**C)** Spectral power of the Fourier-transformed gene expression. The major spectral power at the wavelength of 772 kb is indicated by the broken line in red. (**D)** Periodograms of gene expression. The expression levels (log_10_RPKM) are plotted against the BW25113 genome. The mean expression levels of the genes in every 1-kb sliding window are shown as black lines. The periodicities corresponding to the highest spectral power (**C**, red lines) are shown as the curves in red. The upper and bottom panels indicate the growth in M63 and LB, respectively. The *ori* and *dif* are indicated by broken lines in black. The Fisher’s g test results for the periods in M63 and LB were 0.123 (*p* = 3e − 129) and 0.099 (*p* = 2e − 102), respectively.

To determine the overall patterns/features of the growth rates across the genome, the chromosomal periodicity, which is mediated by genome replication and chromosomal architecture ^29–31^, was evaluated with the Fourier transform method. Both essential and non-essential genes were subjected to the analysis, for which the growth rates with respect to the essential genes were set to zero, as deleting these genes must have led to cell death. The maximal spectral power of the growth rates was exactly the same at the wavelength corresponding to 772 kb in both LB and M63 media (Fig. 5A), resulting in identical chromosomal periodicities of six periods that were independent of the nutritional conditions (Fig. 5B). Comparatively, the chromosomal periodicity of gene expression showed a maximal spectral power at 772 kb (Fig. 5C, red lines), which resulted in a common chromosomal periodicity for gene expression (Fig. 5D, red curves). Of note, the maximal peak at 4,632 kb in LB media was ignored, as it represented the full length of the genome (Fig. 5C). Taken together, the results showed that the chromosomal periodicities of the growth rates (Fig. 5B) and the expression levels (Fig. 5D) were perfectly synchronized in a reverse direction and agreed with the negative correlations between growth and expression (Fig. 2). The conserved six-period periodicity was consistent with our latest finding on the universality of the chromosomal periodicities of *E. coli* transcriptomes^32^. In addition, the gene-independent transcriptional propensity across the *E. coli* genome, which was evaluated by integrating the barcoded reporter constructs^33^, showed the same six-period (Fig. S11), well supporting the universal chromosomal periodicity.

### Hypothesis for the decisive factors determining the common six-period periodicity

The origin of the chromosomal periodicity of the growth rates is an intriguing topic of investigation. The genome architecture was reported to be associated with global gene regulation^34^ and was somehow attributed to nucleoid-associated proteins^31,35–37^. These mechanisms could partially explain the chromosomal periodicity of the transcriptome^32,38–40^, whereas it was inadequate to explain the periodicity of the growth rates of a large number of genetically differentiated strains because the binding activity of nucleotide-associated proteins (NAPs) must have been changed due to genetic disturbances. In addition, by taking into account the chromosomal macrodomain model^41–44^, the conserved six-period periodicity might have been mediated by the four macrodomains and two unstructured regions. However, the chromosomal periods of the growth rates were partially unmatched to the chromosomal macrodomain positions (Fig. S12). In addition to these well-known molecular mechanisms, it is unclear if there is any single mode that determines the common periodicity.

**Figure 6:**
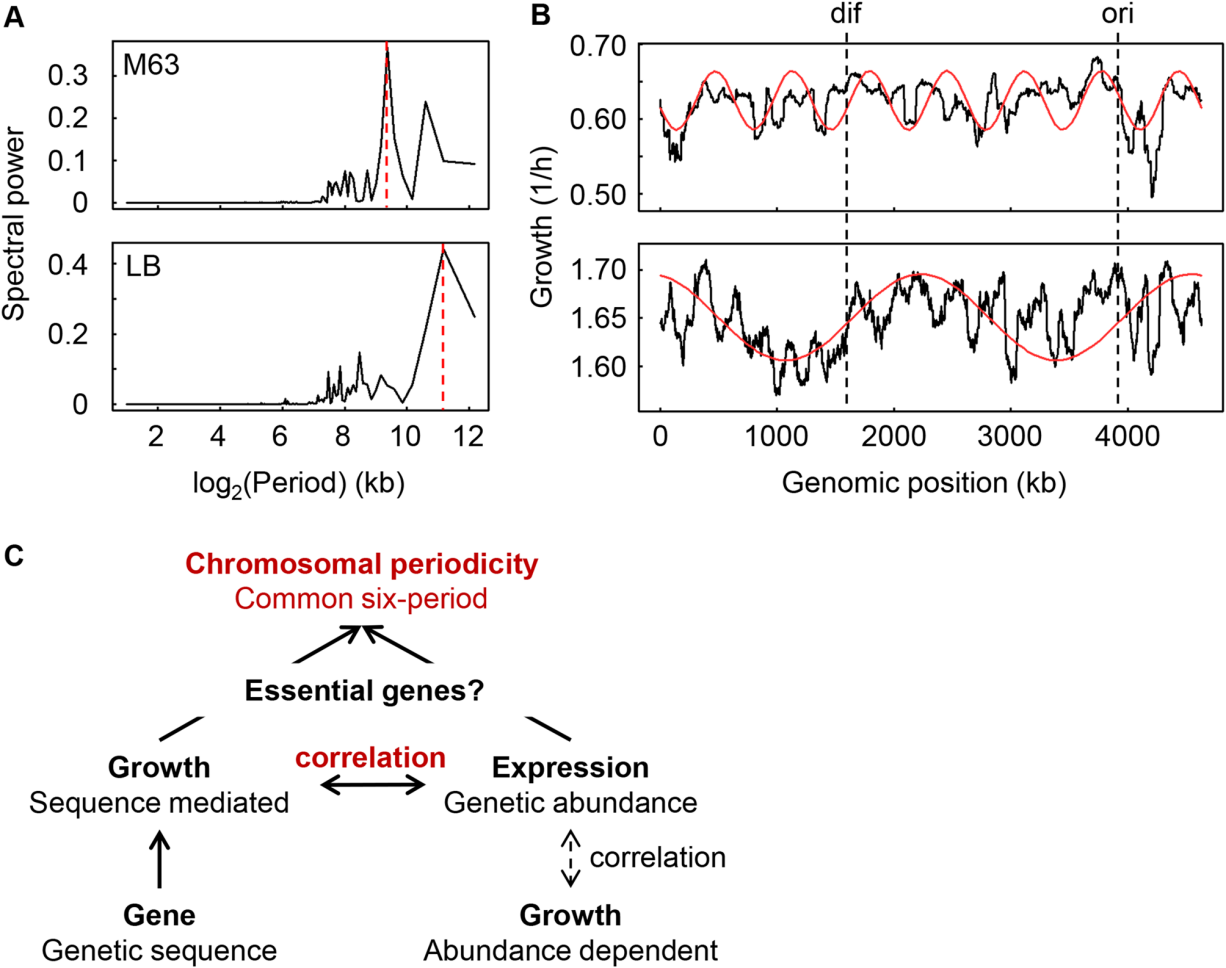
Decision-making factors for the correlated periodicity. **(A)** Spectral power of the Fourier-transformed growth rates of the knockout strains. The broken lines in red indicate the major spectral powers, which were at wavelengths of 661.7 and 2,316 kb in M63 and LB media, respectively. **(B)** Periodograms of the growth rates of the knockout strains. The growth rates of the knockout strains are plotted against the BW25113 genome. The mean growth rates of the strains lacking the corresponding genes (non-essential genes only) in every 1kb sliding window are shown in 100-kb bins by the black lines. The periodicities of the highest spectral power (**A**, red lines) are shown by the red curves. The upper and bottom panels indicate the growth in M63 and LB, respectively. The Fisher’s g test results for the periods in M63 and LB were 0.189 (*p* = 1e − 207) and 0.199 (*p* = 2e − 220), respectively. **(C)** Schematic drawing of the relationships among the genes, their expression and growth. The bold solid and thin broken-line arrows indicate what was found in the present and previous studies, respectively. The novel findings of the global features are highlighted in red.

We assumed that the functional essentiality of the genes contributed to the common periodicity, as the genome organization was connected to the gene function^45^. The periodicity analysis based on the non-essential genes (Keio collection) only resulted in a maximal spectral power at 662 and 2,316 kb (Fig. 6A), which indicated the occurrence of seven and two periods in M63 and LB media, respectively (Fig. 6B). The number of periods was different from the six periods associated with the whole genome (Fig. 5A), which included both essential and nonessential genes. The alteration of the periods strongly suggested that the essential genes contributed to the common six-period periodicity. The correlated chromosomal periodicity of the genetic sequence mediated growth, and the genetic abundance indicated that expression might be mediated by both essential genes (Fig. 6C). It is unknown whether there was any change in the chromosomal periodicity of the transcriptome that lacked the essential genes because it was impossible to experimentally determine the transcriptomes of the essential gene knockout strains. Simply removing the expression data for the essential genes from the wild-type transcriptome dataset did not alter the period^32^. As essential genes are thought to be the most highly conserved genetic information across species, the common periodicities of the growth rates and gene expression might be an evolutionary consequence of the conservation of gene pairs^46^, although the benefit of the six-period periodicity remains unknown.

## Discussion

The present study attempted to link nonessential genes to growth fitness under both rich and poor nutritional conditions to complement the growing pool of knowledge regarding gene functions^25,27,47,48^ and growth models^49–51^. The classification of genes in the fitness category helped us to obtain new insights into the relationships between gene expression and growth fitness. For instance, a large number of genes that were highly expressed in LB media were not classified in the fitness group as having a very positive effect on growth (Figs. 1, 2, 3), which indicated that the upregulated genes in enriched conditions did not contribute to fitness in either rich (LB) or poor (M63) nutritional conditions. Intriguingly, comparing the growth rates of the single-gene knockout strains to those of the genome-reduced strains^13^ revealed that the contribution of genetic information to growth fitness was somehow additive in a nutritionally dependent manner. The mean growth rates of the knockout strains lacking the genes located within the deleted genome regions were weakly correlated with the growth rates of the genome-reduced strains lacking the corresponding genome regions in LB media, but there was no significant correlation in cells grown in M63 media (Fig. S13). This result indicated that the genetic contribution to growth fitness was additive in enriched conditions but nonadditive in poor nutritional conditions, which was probably due to the different requirements of metabolism and gene function in LB and M63 media.

A significant correlation between the growth rates of the single-gene knockout strains and the original expression levels of these genes in their parental strain was identified for the first time. In particular, the gene deficiency-related growth rates presented the same chromosomal periodicity as that of the wild-type transcriptome. By taking into account the previous findings on the periodicity of mutation rates in bacterial genomes^31^ and the correlation between mutation rates and growth rates^52^, we assumed that the global parameters of living organisms (*e.g.*, growth rate, mutation rate, transcriptome, genome, etc*.*) were correlated to each other in a chromosomally periodic manner. The genomic position of the gene was linked to its expression level^53,54^ and the timing of expression^55^, which was due to chromosomal organization^56^. The chromosome was structured by the interactions between ongoing replication and transcription in exponentially growing cells^57^, which was reflected by the growth rate. Thus, the global parameters were coordinated. As the chromosomal periodicity in gene expression^32,38–40^ was explained by the chromosomal macrodomains^42–44^, the common correlated chromosomal periodicities of the growth rates and gene expression might be a general feature governing the growth of cells.

A positive correlation between the growth rate and the growth maximum was significantly detected and was dependent on the growth media (Fig. S14). This conflicted somewhat with the r/K selection mechanism^22,58^, which was often found to represent the trade-off between the growth rate and the population size^59–61^. To verify the correlation, we reanalysed three public data sets reporting the growth profiles of the strains of the Keio collection. The correlation of the growth rate and the growth maximum was commonly observed (Fig. S15) regardless of the variation in assay methodologies, media and growth mode used in these studies^3–5^. It was unclear whether the positive correlation was due to any experimental restriction common to the high-throughput growth assays used in these studies. It is of note that the growth assay performed in microwell plates in a plate reader could lead to locational biases^3,62^. As the replicates were performed in different well locations, the operational bias could be ignored. The mean values of the growth rates of different strains acquired from the same well were random, but the maximal OD_600_ values changed gradually in a manner dependent on the well location (Fig. S16), which is consistent with previous findings^62^. The locational bias was independent of the correlation of the growth rate, and the growth maximum seemed to be universal and was in line with the observation of a trade-off within but not between populations in terms of experimental evolution^63^.

In summary, in comparison to previous studies that successfully coupled phenotypes to individual genes from a systematic point of view^64,65^, the present study directly connected the growth fitness to individual genes and their expression levels as part of a global pattern, *i.e.*, the correlated chromosomal periodicity. The working mechanism responsible for the common periodicity has remained a black box, although the essential genes might play a role. The present study provided a valuable data set and periodic rule for growth prediction regarding the minimal genome, which has been discussed for decades^66–68^ and experimentally challenged by reducing genomic redundancy^69,70^ and synthetic reconstruction^71,72^ to preserve essential metabolism^73^.

## Materials and methods

### Strains and media

A total of 3,909 single-gene knockout strains (the Keio collection) and the wild-type strain BW25113, which was used to construct the Keio collection, were obtained from the National Institute of Genetics in Japan. These strains were routinely grown in nutrient-rich Luria–Bertani (LB) and M63 minimal media, which were described previously in detail^13,62^.

### Storage of the Keio collection for the repeated growth assays

The Keio collection (3,909 strains) was inoculated from agar plates into 96-well stock plates containing 200 μl per well of LB or M63 medium and incubated overnight at 37 °C without shaking. The overnight cultures were used for stock. Fifty microlitres of 60% glycerol was added to each well, and the plate was stored at − 80 °C. Those wells that showed no growth after overnight incubation were inoculated again into 3 ml LB medium in tubes and cultured with shaking. After the strains showed growth, 60% glycerol was added, and the tubes were stored at − 80 °C. The wild-type strain BW25113 was inoculated into 6 ml of LB medium and grown for 6 h at 37 °C with shaking. The resulting cell cultures were stored at − 80 °C in frozen tubes containing 25% glycerol.

### High-throughput growth assay

The *E. coli* strains were grown in both LB and M63 media as previously described^13^. The strains were inoculated from the glycerol stock into new 96-well microplates (Corning), where each well contained 200 μl of media. The 96-well microplates were incubated in a plate reader with a rotation rate of 567 cpm at 37 °C. Temporal growth was detected at an absorbance of 600 nm, and readings were obtained at 15- or 30-min intervals for 24 to 48 h. Three independent growth assays were performed for each strain in each medium.

### Calculation of the growth rate

Data from the plate reader were acquired with Gen5 software and then exported as Excel files. The growth rates during the exponential phase were calculated with the following equation as previously reported^62^.

1 $$\upmu =\frac{\mathrm{LN}({\mathrm{C}}_{\mathrm{i}+1}/{\mathrm{C}}_{\mathrm{i}})}{{\mathrm{t}}_{\mathrm{i}+1}-{\mathrm{t}}_{\mathrm{i}}}$$

Here, C_i_ and C_i+1_ represent the two readings of the OD_600_ values at two continuous time points of t_i_ and t_i+1_, which were obtained at intervals of 15 or 30 min. The five continuous growth rates that exhibited the largest means and the smallest standard deviations were averaged to determine the growth rate (h^−1^). The average values and standard errors derived from repeated tests were calculated.

### Total RNA purification

Three independent cell cultures were used for RNAseq to determine the mean expression of genes under each culture condition. Cell culture was performed in 5 mL test tubes, and cell collection and purification of the total RNA were performed. The *E. coli* cells were inoculated into fresh LB and M63 media at 37 °C. Triplicate cultures were grown for 2 h until an OD_600_ value of 0.01 ~ 0.1 was reached in the test tubes. Samples were withdrawn and immediately mixed with 5 ml of RNA stop solution (90% ethanol and 10% phenol) on ice. Cells were pelleted by centrifugation (7,000 rpm, 3 min) and frozen at − 80 °C. The stocked pellets were subsequently thawed on ice and immediately processed for RNA extraction using the RNeasy Mini Kit (QIAGEN) according to the manufacturer’s instructions. The total RNA was resuspended in RNase-free water and stored at − 80 °C.

### RNAseq

Whole transcriptome sequencing of wild-type *E. coli* BW25113 was performed with the NovaSeq Sequencer (Illumina) with the sequencing control software 1000000019358 v02. The rRNAs were removed using the ribo-zero RNA removal Kit for gram-negative bacteria (Illumina). mRNA preparation was performed with the TruSeq Stranded mRNA LT sample prep kit in accordance with the TruSeq Stranded mRNA sample preparation guide (part #15031047 rev. E). The NovaSeq 6000 S4 reagent kit was used for sequencing according to the NovaSeq 6000 system user guide document #1000000019358 v02. The trimmed reads were mapped to the reference genome sequence of *Escherichia coli BW25113 GCF_000750555.1_ASM75055v1* with Bowtie software. The raw RNAseq data sets were deposited into the NCBI Gene Expression Omnibus database under the GEO Series accession number GSE136101.

### Transcriptome analyses

The datasets with raw expression data were subjected to global normalization, resulting in a common median value (logarithmic value, log_10_RPKM) for all data sets as previously described^74,75^. The mean values of three biological replicates were used for the following analyses. All statistical tests and computational analyses were performed using either R^76^ or Mathematica 11 (MathWorks). The differentially expressed genes (DEGs) and the differentiated growth rates (DGGs) were identified according to the rank product^77,78^, and the significance of the functional enrichment based on the gene category^27^ was evaluated by binomial tests with the Bonferroni correction, as previously reported^21,74^.

### Evaluation of chromosomal periodicity

Chromosomal periodicity was calculated using a standard Fourier transform method with a sliding distance of 1kb and 100-kb bins as previously described^79^. A standard Fourier transform method was used for the determination of chromosomal periodicity. The significance of the periodicity was assessed with Fisher’s g test^80^. The approximate line corresponding to the periodicity was calculated based on the highest peak (statistical significance) of the periodogram and was fitted by minimizing the squared error between the approximate line and the series of expression values. The genomic positions of *ori*, *dif*, and the chromosomal macrodomains were determined as referred to in previous reports^43,56^.

## Supplementary information

Supplementary Information.

## References

[1] Joyce AR (2006). Experimental and computational assessment of conditionally essential genes in *Escherichia coli*. J. Bacteriol..

[2] Baba, T. *et al.* Construction of *Escherichia coli* K-12 in-frame, single-gene knockout mutants: the Keio collection. *Mol. Syst. Biol.* **2**, 0008, 10.1038/msb4100050 (2006). 10.1038/msb4100050 PMC1681482 16738554

[3] Falls KC, Williams AL, Bryksin AV, Matsumura I (2014). *Escherichia coli* deletion mutants illuminate trade-offs between growth rate and flux through a foreign anabolic pathway. PLoS ONE.

[4] Takeuchi R (2014). Colony-live-a high-throughput method for measuring microbial colony growth kinetics-reveals diverse growth effects of gene knockouts in *Escherichia coli*. BMC Microbiol..

[5] Campos M (2018). Genomewide phenotypic analysis of growth, cell morphogenesis, and cell cycle events in *Escherichia coli*. Mol. Syst. Biol..

[6] Ursell T (2017). Rapid, precise quantification of bacterial cellular dimensions across a genomic-scale knockout library. BMC Biol..

[7] French, S., Cote, J. P., Stokes, J. M., Truant, R. & Brown, E. D. Bacteria getting into shape: Genetic determinants of *E. coli* morphology. *mBio* **8**, e01977–01916, 10.1128/mBio.01977-16 (2017). 10.1128/mBio.01977-16 PMC5340871 28270582

[8] Guzman GI (2018). Reframing gene essentiality in terms of adaptive flexibility. BMC Syst. Biol..

[9] Cote, J. P. *et al.* The genome-wide interaction network of nutrient stress genes in *Escherichia coli*. *mBio* **7**, e01714–01716, 10.1128/mBio.01714-16 (2016). 10.1128/mBio.01714-16 PMC5120140 27879333

[10] Posfai G (2006). Emergent properties of reduced-genome *Escherichia coli*. Science.

[11] Kato J, Hashimoto M (2007). Construction of consecutive deletions of the Escherichia coli chromosome. Mol. Syst. Biol..

[12] Mizoguchi H, Sawano Y, Kato J, Mori H (2008). Superpositioning of deletions promotes growth of *Escherichia coli* with a reduced genome. DNA Res..

[13] Kurokawa M, Seno S, Matsuda H, Ying BW (2016). Correlation between genome reduction and bacterial growth. DNA Res..

[14] Karcagi I (2016). Indispensability of horizontally transferred genes and its impact on bacterial genome streamlining. Mol. Biol. Evol..

[15] Hashimoto M (2005). Cell size and nucleoid organization of engineered *Escherichia coli* cells with a reduced genome. Mol. Microbiol..

[16] Scott M, Gunderson CW, Mateescu EM, Zhang Z, Hwa T (2010). Interdependence of cell growth and gene expression: Origins and consequences. Science.

[17] Sastry AV (2019). The *Escherichia coli* transcriptome mostly consists of independently regulated modules. Nat. Commun..

[18] Guell M, Yus E, Lluch-Senar M, Serrano L (2011). Bacterial transcriptomics: What is beyond the RNA horizome?. Nat. Rev. Microbiol..

[19] Feugeas JP (2016). Links between transcription, environmental adaptation and gene variability in *Escherichia coli*: Correlations between gene expression and gene variability reflect growth efficiencies. Mol. Biol. Evol..

[20] Nahku R (2010). Specific growth rate dependent transcriptome profiling of *Escherichia coli* K12 MG1655 in accelerostat cultures. J. Biotechnol..

[21] Matsumoto Y, Murakami Y, Tsuru S, Ying BW, Yomo T (2013). Growth rate-coordinated transcriptome reorganization in bacteria. BMC Genomics.

[22] Weisse AY, Oyarzun DA, Danos V, Swain PS (2015). Mechanistic links between cellular trade-offs, gene expression, and growth. Proc. Natl. Acad. Sci. USA.

[23] Jozefczuk S (2010). Metabolomic and transcriptomic stress response of *Escherichia coli*. Mol. Syst. Biol..

[24] Lopez-Maury L, Marguerat S, Bahler J (2008). Tuning gene expression to changing environments: From rapid responses to evolutionary adaptation. Nat. Rev. Genet..

[25] 25Salgado, H. *et al.* RegulonDB v8.0: Omics data sets, evolutionary conservation, regulatory phrases, cross-validated gold standards and more. *Nucleic Acids Res.* **41**, D203–213, 10.1093/nar/gks1201 (2013). 10.1093/nar/gks1201 PMC3531196 23203884

[26] Fang X (2017). Global transcriptional regulatory network for *Escherichia coli* robustly connects gene expression to transcription factor activities. Proc. Natl. Acad. Sci. USA.

[27] Riley M (2006). *Escherichia coli* K-12: A cooperatively developed annotation snapshot-2005. Nucleic Acids Res..

[28] Love MI, Huber W, Anders S (2014). Moderated estimation of fold change and dispersion for RNA-seq data with DESeq2. Genome Biol..

[29] Lal A (2016). Genome scale patterns of supercoiling in a bacterial chromosome. Nat. Commun..

[30] Krogh TJ, Moller-Jensen J, Kaleta C (2018). Impact of chromosomal architecture on the function and evolution of bacterial genomes. Front. Microbiol..

[31] Dillon SC, Dorman CJ (2010). Bacterial nucleoid-associated proteins, nucleoid structure and gene expression. Nat. Rev. Microbiol..

[32] Nagai, M., Kurokawa, M. & Ying, B. W. The highly conserved chromosomal periodicity of transcriptomes and the correlation of its amplitude with the growth rate in *Escherichia coli*. *DNA Res*. dsaa018. 10.1093/dnares/dass018 (2020). 10.1093/dnares/dsaa018 PMC7508348 32866232

[33] Scholz SA (2019). High-Resolution mapping of the *Escherichia coli* chromosome reveals positions of high and low transcription. Cell Syst.

[34] Dorman CJ (2013). Genome architecture and global gene regulation in bacteria: Making progress towards a unified model?. Nat. Rev. Microbiol..

[35] Lioy, V. S. *et al.* Multiscale structuring of the *E. coli* chromosome by nucleoid-associated and condensin proteins. *Cell* **172**, 771–783 e718, 10.1016/j.cell.2017.12.027 (2018). 10.1016/j.cell.2017.12.027 29358050

[36] Wang W, Li GW, Chen C, Xie XS, Zhuang X (2011). Chromosome organization by a nucleoid-associated protein in live bacteria. Science.

[37] Browning DF, Grainger DC, Busby SJ (2010). Effects of nucleoid-associated proteins on bacterial chromosome structure and gene expression. Curr. Opin. Microbiol..

[38] Jeong KS, Ahn J, Khodursky AB (2004). Spatial patterns of transcriptional activity in the chromosome of *Escherichia coli*. Genome Biol..

[39] Postow L, Hardy CD, Arsuaga J, Cozzarelli NR (2004). Topological domain structure of the *Escherichia coli* chromosome. Genes Dev..

[40] Allen TE, Price ND, Joyce AR, Palsson BO (2006). Long-range periodic patterns in microbial genomes indicate significant multi-scale chromosomal organization. PLoS Comput. Biol..

[41] Espeli, O., Mercier, R. & Boccard, F. DNA dynamics vary according to macrodomain topography in the *E. coli* chromosome. *Mol. Microbiol.* **68**, 1418–1427, 10.1111/j.1365-2958.2008.06239.x (2008). 10.1111/j.1365-2958.2008.06239.x 18410497

[42] Niki H, Yamaichi Y, Hiraga S (2000). Dynamic organization of chromosomal DNA in *Escherichia coli*. Genes Dev..

[43] Valens M, Penaud S, Rossignol M, Cornet F, Boccard F (2004). Macrodomain organization of the *Escherichia coli* chromosome. EMBO J..

[44] Boccard F, Esnault E, Valens M (2005). Spatial arrangement and macrodomain organization of bacterial chromosomes. Mol. Microbiol..

[45] Slager J, Veening JW (2016). Hard-wired control of bacterial processes by chromosomal gene location. Trends Microbiol..

[46] Wright MA, Kharchenko P, Church GM, Segre D (2007). Chromosomal periodicity of evolutionarily conserved gene pairs. Proc. Natl. Acad. Sci. USA..

[47] Keseler IM (2017). The EcoCyc database: Reflecting new knowledge about *Escherichia coli* K-12. Nucleic Acids Res..

[48] Kanehisa M, Sato Y, Kawashima M, Furumichi M, Tanabe M (2016). KEGG as a reference resource for gene and protein annotation. Nucleic Acids Res..

[49] O'Brien EJ, Lerman JA, Chang RL, Hyduke DR, Palsson BO (2013). Genome-scale models of metabolism and gene expression extend and refine growth phenotype prediction. Mol. Syst. Biol..

[50] Goh S, Boberek JM, Nakashima N, Stach J, Good L (2009). Concurrent growth rate and transcript analyses reveal essential gene stringency in *Escherichia coli*. PLoS ONE.

[51] Swain PS (2016). Inferring time derivatives including cell growth rates using Gaussian processes. Nat. Commun..

[52] Nishimura, I., Kurokawa, M., Liu, L. & Ying, B. W. Coordinated changes in mutation and growth rates induced by genome reduction. *mBio* **8**, 10.1128/mBio.00676-17 (2017). 10.1128/mBio.00676-17 PMC5573674 28679744

[53] Couturier E, Rocha EP (2006). Replication-associated gene dosage effects shape the genomes of fast-growing bacteria but only for transcription and translation genes. Mol. Microbiol..

[54] Ying BW, Tsuru S, Seno S, Matsuda H, Yomo T (2014). Gene expression scaled by distance to the genome replication site. Mol. BioSyst..

[55] Sobetzko P, Travers A, Muskhelishvili G (2012). Gene order and chromosome dynamics coordinate spatiotemporal gene expression during the bacterial growth cycle. Proc. Natl. Acad. Sci. USA.

[56] Bryant JA, Sellars LE, Busby SJ, Lee DJ (2014). Chromosome position effects on gene expression in *Escherichia coli* K-12. Nucleic Acids Res..

[57] Cagliero C, Grand RS, Jones MB, Jin DJ, O'Sullivan JM (2013). Genome conformation capture reveals that the *Escherichia coli* chromosome is organized by replication and transcription. Nucleic Acid Res..

[58] Engen S, Lande R, Saether BE (2013). A quantitative genetic model of r- and K-selection in a fluctuating population. Am. Nat..

[59] Bachmann H (2013). Availability of public goods shapes the evolution of competing metabolic strategies. Proc. Natl. Acad. Sci. USA.

[60] Ying BW (2015). Evolutionary consequence of a trade-off between growth and maintenance along with ribosomal damages. PLoS ONE.

[61] Manhart M, Shakhnovich EI (2018). Growth tradeoffs produce complex microbial communities on a single limiting resource. Nat. Commun..

[62] Kurokawa M, Ying BW (2017). Precise, high-throughput analysis of bacterial growth. J. Vis. Exp..

[63] Novak, M., Pfeiffer, T., Lenski, R. E., Sauer, U. & Bonhoeffer, S. Experimental tests for an evolutionary trade-off between growth rate and yield in *E. coli*. *Am. Nat.* **168**, 242–251, 10.1086/506527 (2006). 10.1086/506527 16874633

[64] Nichols RJ (2011). Phenotypic landscape of a bacterial cell. Cell.

[65] Galardini, M. *et al.* Phenotype inference in an *Escherichia coli* strain panel. *Elife* **6**, 10.7554/eLife.31035 (2017). 10.7554/eLife.31035 PMC5745082 29280730

[66] Gil, R., Silva, F. J., Pereto, J. & Moya, A. Determination of the core of a minimal bacterial gene set. *Microbiol. Mol. Biol. Rev.* **68**, 518–537 (table of contents), 10.1128/MMBR.68.3.518-537.2004 (2004). 10.1128/MMBR.68.3.518-537.2004 PMC515251 15353568

[67] Martinez-Garcia E, de Lorenzo V (2016). The quest for the minimal bacterial genome. Curr. Opin. Biotechnol..

[68] Xavier JC, Patil KR, Rocha I (2014). Systems biology perspectives on minimal and simpler cells. Microbiol. Mol. Biol. Rev..

[69] Feher T, Papp B, Pal C, Posfai G (2007). Systematic genome reductions: Theoretical and experimental approaches. Chem. Rev..

[70] Kurokawa M, Ying BW (2019). Experimental challenges for reduced genomes: The cell model *Escherichia coli*. Microorganisms.

[71] Jewett MC, Forster AC (2010). Update on designing and building minimal cells. Curr. Opin. Biotechnol..

[72] Hutchison, C. A., 3rd *et al.* Design and synthesis of a minimal bacterial genome. *Science* **351**, aad6253, 10.1126/science.aad6253 (2016). 10.1126/science.aad6253 27013737

[73] Breuer, M. *et al.* Essential metabolism for a minimal cell. *Elife* **8**, 10.7554/eLife.36842 (2019). 10.7554/eLife.36842 PMC6609329 30657448

[74] Ying BW, Yama K (2018). Gene expression order attributed to genome reduction and the steady cellular state in *Escherichia coli*. Front. Microbiol..

[75] Ying BW (2015). Bacterial transcriptome reorganization in thermal adaptive evolution. BMC Genomics.

[76] Ihaka R, Gentleman R (1996). R: A language for data analysis and graphics. J. Comput. Graph. Stat..

[77] Breitling R, Armengaud P, Amtmann A, Herzyk P (2004). Rank products: a simple, yet powerful, new method to detect differentially regulated genes in replicated microarray experiments. FEBS Lett..

[78] Hong F (2006). RankProd: A bioconductor package for detecting differentially expressed genes in meta-analysis. Bioinformatics.

[79] Ying BW, Seno S, Kaneko F, Matsuda H, Yomo T (2013). Multilevel comparative analysis of the contributions of genome reduction and heat shock to the *Escherichia coli* transcriptome. BMC Genomics.

[80] Wichert S, Fokianos K, Strimmer K (2004). Identifying periodically expressed transcripts in microarray time series data. Bioinformatics.

