# Supplementary material for: Correlated chromosomal periodicities according to the growth rate and gene expression
Source: Sci Rep. 2020 Sep 23;10:15531. doi: 10.1038/s41598-020-72389-6 (PMC7511328; doi:10.1038/s41598-020-72389-6)
Supplement: Supplementary file 1 — Supplementary Information. [file 41598_2020_72389_MOESM1_ESM.pdf]

Supplemental figures and tables

Correlated chromosomal periodicities according to the growth rate and gene expression

Liu Liu[1,#], Masaomi Kurokawa[1,#], Motoki Nagai[1], Shigeto Seno[2], Bei-Wen Ying[1,*]

[1] Graduate School of Life and Environmental Sciences, University of Tsukuba, 1-1-1 Tennodai, Tsukuba, Ibaraki 305-8572, Japan
[2] Graduate School of Information Science and Technology, Osaka University, 1-5 Yamadaoka, Suita, Osaka 565-0871, Japan

[#] These authors contributed equally to the study.

[*]Corresponding: ying.beiwen.gf@u.tsukuba.ac.jp (BWY)

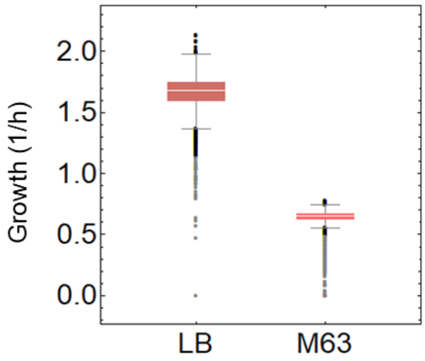

Figure S1 Boxplots of the growth rates of the single-gene knockout strains. The growth rates of a total of 3909 single-gene knockout strains grown in LB and M63 media are shown in the box-and-whisker plots. The outliers are indicated.

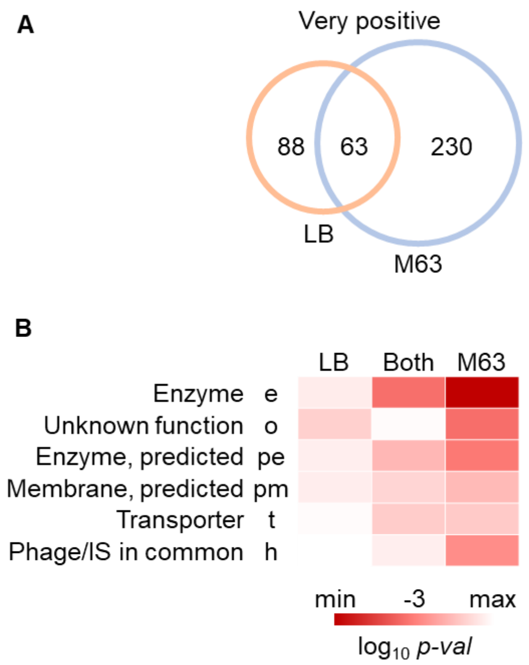

Figure S2 Genes classified as very positive. A. Venn diagram of the genes determined to have a very positive contribution to growth. The numbers of genes (strains) identified specifically and in common in cells grown in LB and M63 are indicated. **B.** Heatmap of the enriched gene categories of the genes determined to have a very positive contribution to growth. The gradation from light to dark indicates the statistical significance of the enrichment analysis from low to high, respectively.

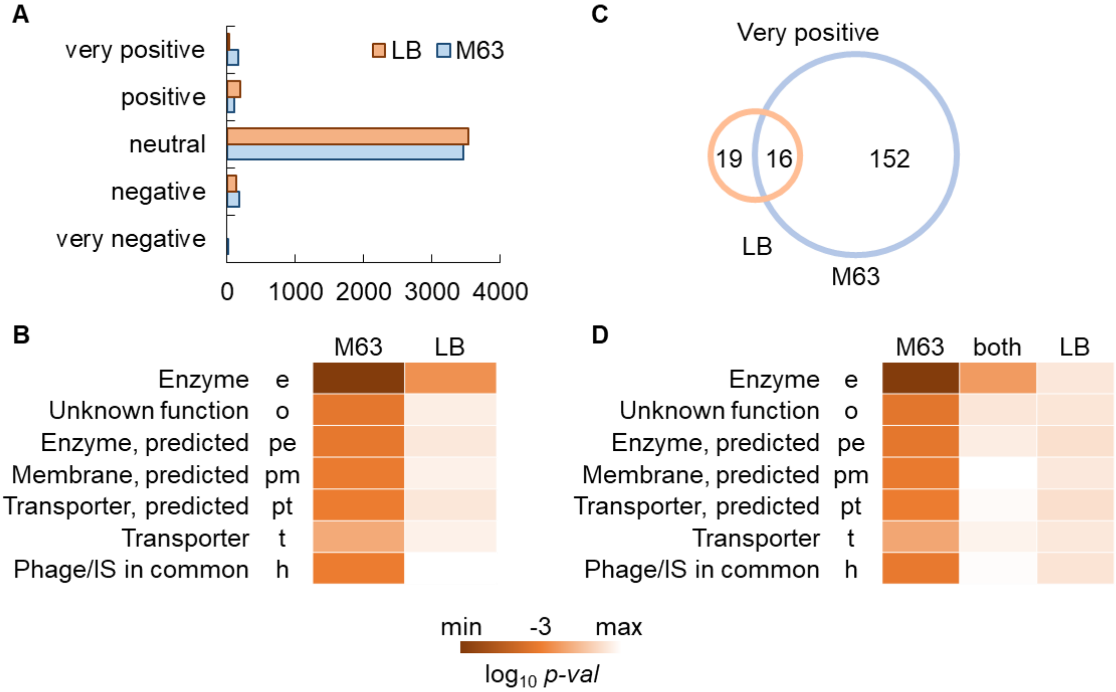

Figure S3 Gene classification and functional enrichment. A. Gene classification based on the growth rate with statistical re-evaluation. Five gene classes (very negative, negative, neutral, positive, and very positive) defined in the LB and M63 media are shown in orange and blue, respectively, as described in Fig. 1. The additional student t test (one-tailed) were performed to all strains, to evaluate whether the mean growth rate of each strain, which was assayed for three times, was statistically within the ranges of the outliers greater than the 1.5 interquartile range (IQR), from the 1.5 IQR to the upper quartile, from the upper to the lower quartiles, from the lower quartile to the 1.5 IQR, and within the ranges of outliers lower than 1.5 IQR, respectively. **B.** Heatmap of the enriched gene categories for the very positive class. The enriched gene categories, which were based on the critical benchmark, were as similar as those shown in Fig. 1C. The gradation from light to dark orange indicates the statistical significance of the enrichment analysis from low to high, respectively. **C.** Venn diagram of the genes determined to have a very positive contribution to growth. According to the critical benchmark, the numbers of genes (strains), identified specifically and in common in cells grown in LB and M63 are indicated. **D.** Heatmap of the enriched gene categories of the genes determined to have a very positive contribution to growth. The enriched gene categories, which were based on the critical benchmark, were as similar as those shown in Fig. S2B. The gradation from light to dark orange indicates the statistical significance of the enrichment analysis from low to high, respectively.

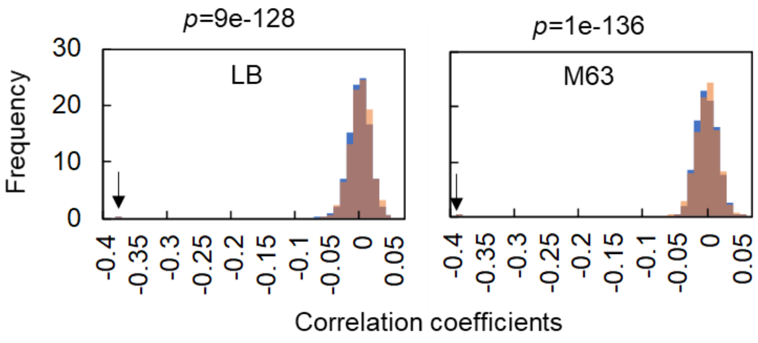

Figure S4 Histograms of the simulated correlation coefficients. The correlation coefficients were simulated by randomizing the values of either the growth rates or the gene expression 1,000 times. The 1,000 correlation coefficients for the randomized growth rates that were simulated and the randomized gene expression are shown in the histograms in blue and orange, respectively. The simulations were performed on the data sets acquired from cells grown in both LB (left) and M63 (right) media. The *p*-values of the correlation coefficients determined in the experiments in comparison to the simulations are indicated.

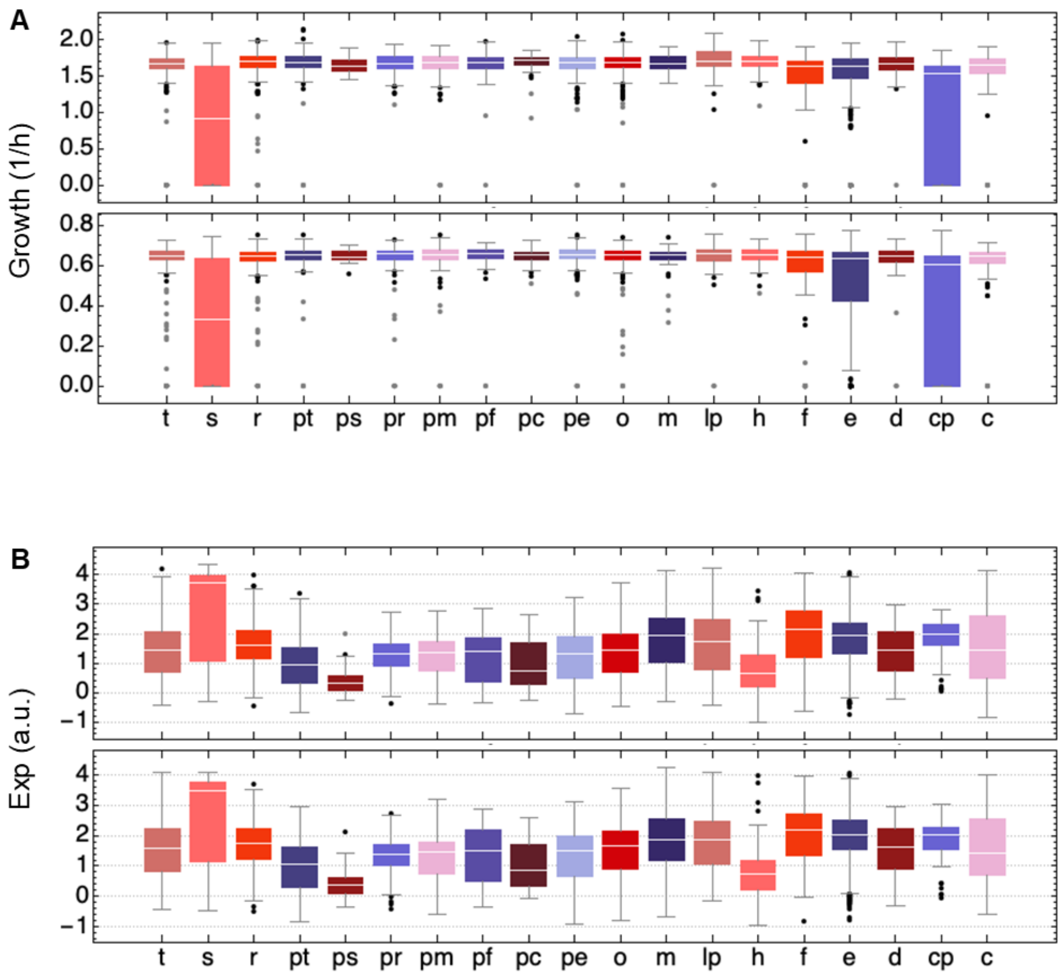

Figure S5 Boxplots of the growth rates and gene expression at the gene category level. The growth rates (**A**) and the expression levels ($\log_{10}$RPKM) (**B**) of the genes assigned to the same gene category are summarized in the box-and-whisker plots. The outliers are indicated. A total of 19 gene categories, which contain more than 30 genes, are shown. The gene categories are indicated as abbreviations.

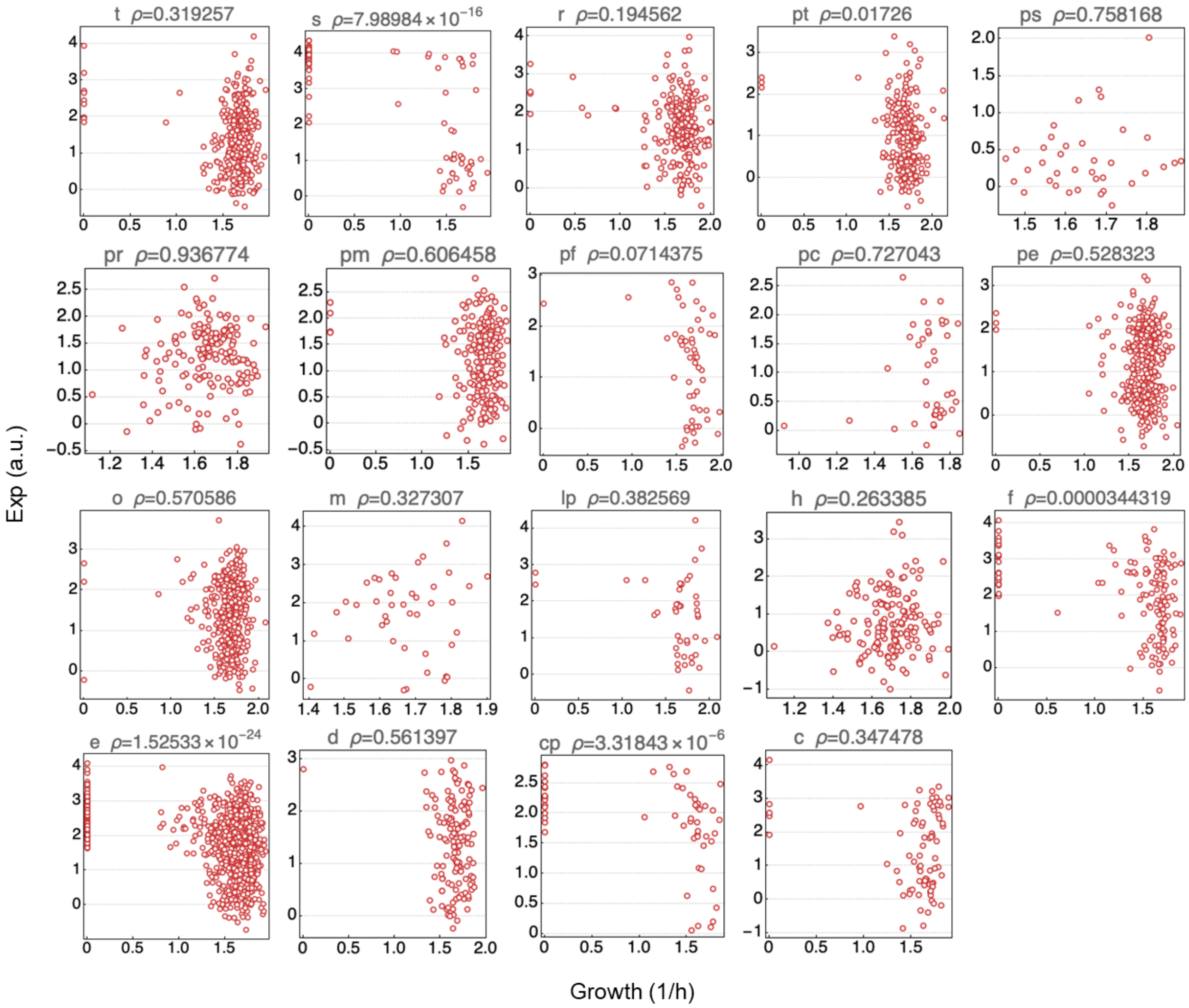

Figure S6 Scatter plots of the growth rates and gene expression in LB within each gene category. The growth rates and the expression levels ($\log_{10}$RPKM) of the genes assigned to the corresponding gene categories are plotted. A total of 19 gene categories, which contain more than 30 genes, are shown. The statistical significance of the Spearman correlation (ρ) is indicated.

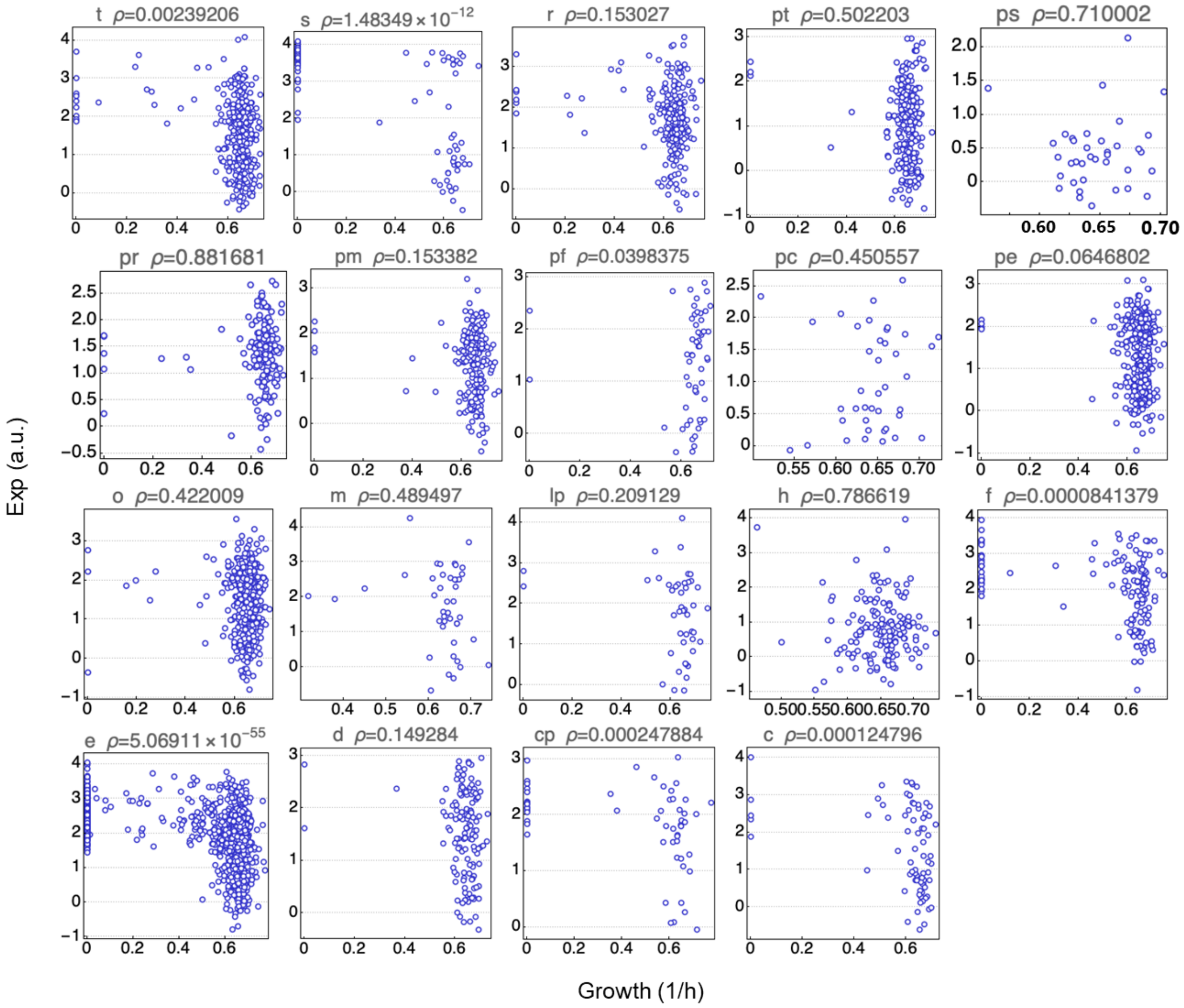

Figure S7 Scatter plots of the growth rates and the gene expression in M63 within each gene category. The growth rates and the expression levels ($\log_{10}$RPKM) of the genes assigned to the corresponding gene categories are plotted. A total of 19 gene categories, which contain more than 30 genes, are shown. The statistical significance of the Spearman correlation (ρ) is indicated.

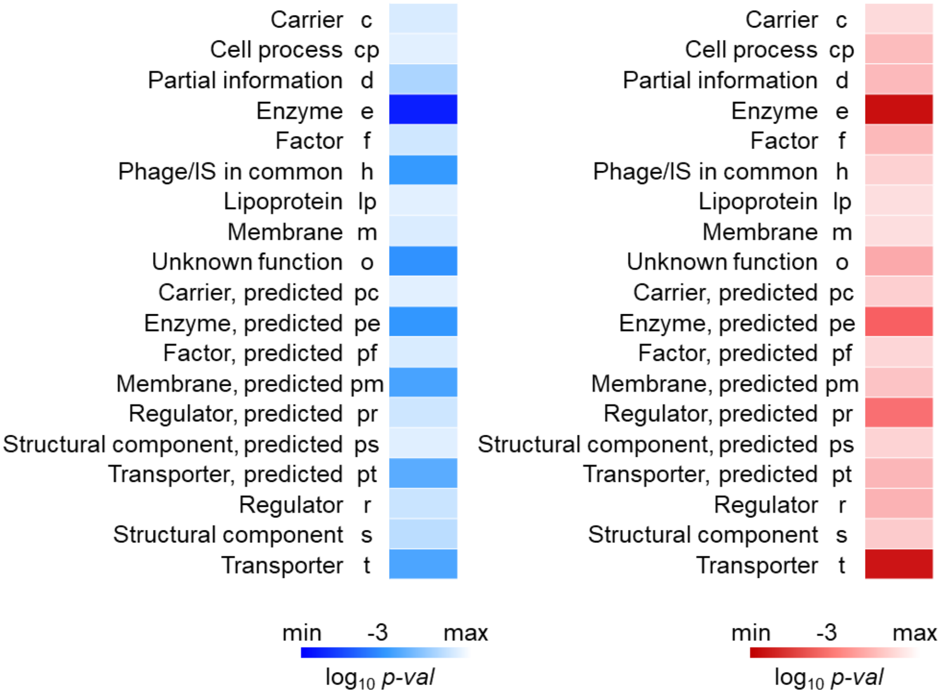

Figure S8 Heatmaps of the enriched gene categories. The full results of the gene category enrichment of DEGs and DGGs are shown in the right and left panels, respectively. The blue and red gradations from light to dark indicate the statistical significance of the enrichment analysis from low to high of the DGGs and DEGs, respectively.

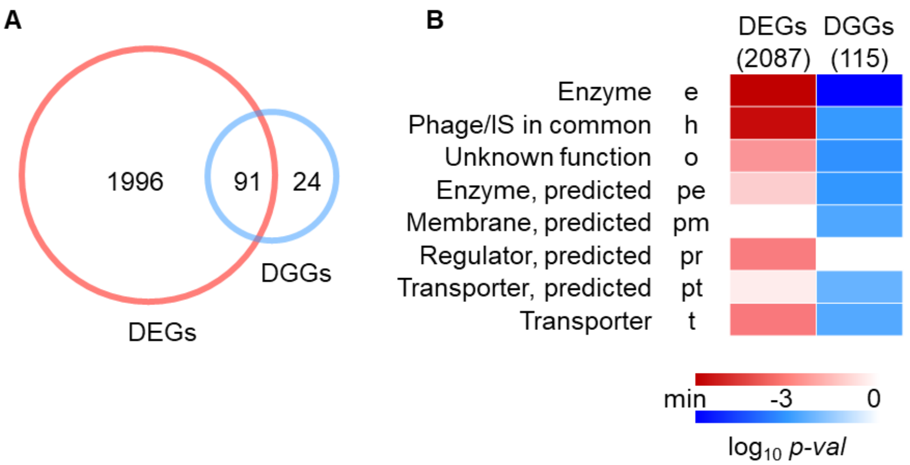

Figure S9 Genes classified as very positive. A. Venn diagram of DEGs and DGGs. The numbers of the genes specifically and commonly determined as DEGs and DGGs are indicated. The methods of DEseq2 and Rank product were used to determine DEGs and DGGs, respectively. **B.** Heatmap of the enriched gene categories of the genes determined to have a very positive contribution to growth. The gradation from light to dark indicates the statistical significance of the enrichment analysis from low to high, respectively.

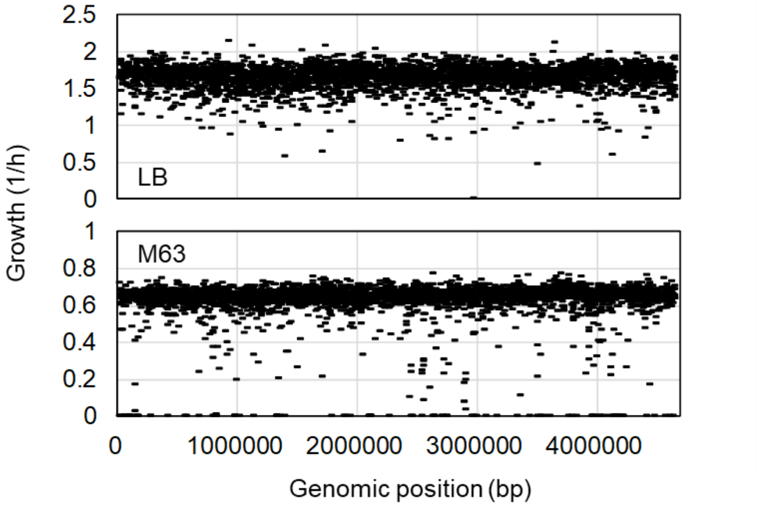

Figure S10 Genomic views of the growth rates of the knockout strains. The growth rates of the knockout strains are directly plotted against those of the wild-type genome BW25113 at the genomic positions of the corresponding missing genes. The upper and bottom panels present the growth rates in LB and M63 media, respectively.

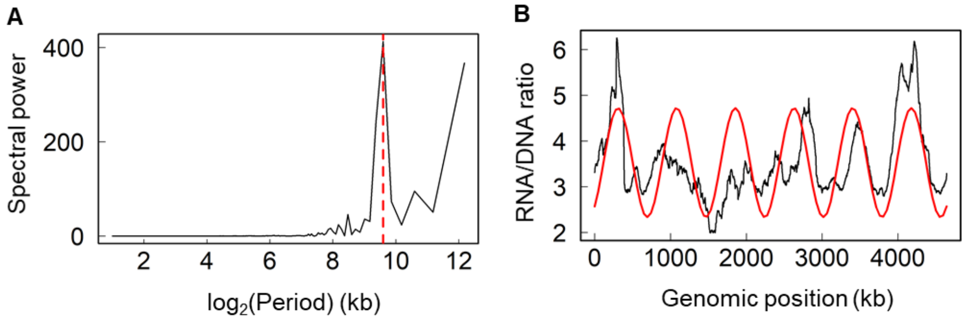

Figure S11 Chromosomal periodicity of the gene-independent transcriptional propensity. **A.** Spectral power of the Fourier-transformed transcriptional propensity. The major spectral power at a wavelength of 773.7 kb is indicated by the broken line in red. **B.** Periodogram of the transcriptional propensity. The periodicities of the highest spectral power (**A**, red lines) are shown in the curves in red. The data of transcriptional propensity was from the previous report[32].

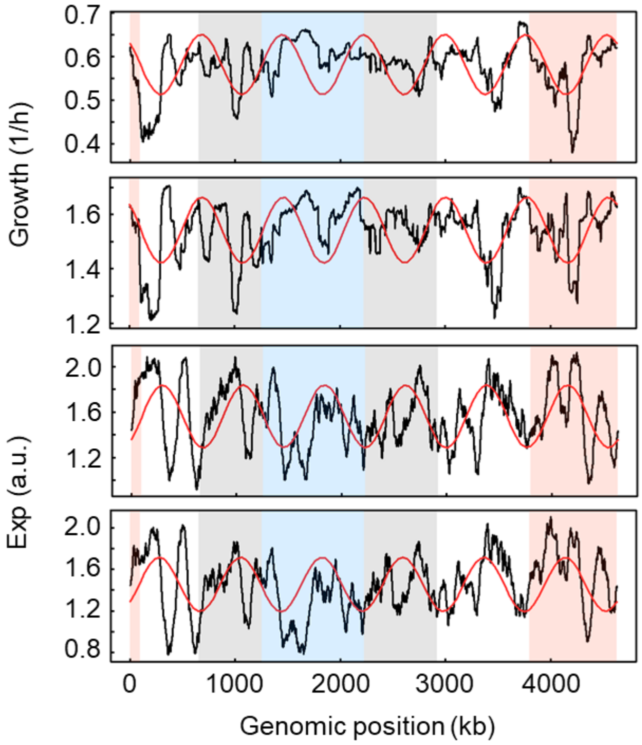

Figure S12 Periodograms of the growth and gene expression associated with the macrodomain distribution. The periodograms in Figure 5B and 5D associated with the chromosomal macrodomains are shown in the upper and bottom panels. The four structured macrodomains of *ori*, right, *ter,* left, are shown in pink, grey, blue and grey, respectively. The two non-structured regions at the right and left (NSR and NSL) are transparent.

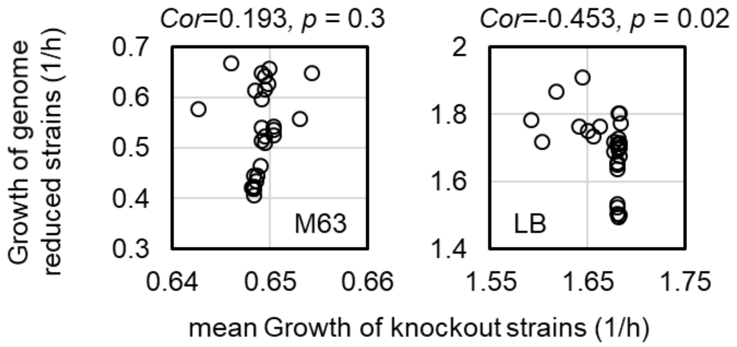

Figure S13 Scatter plots of the mean growth rates of the genome-reduced and single-gene knockout strains. The growth rates of 28 genome-reduced strains, which were constructed from wild-type *E. coli* W3110, were cited from our previous study. The growth rates of the knockout strains missing the genes located within the deleted regions of the reduced genomes were averaged. The growth assays of the genome-reduced strains and the single-gene knockout strains were performed by using the exactly same method. The left and right panels represent growth in M63 and LB media, respectively. The correlation coefficients and the statistical significance are indicated.

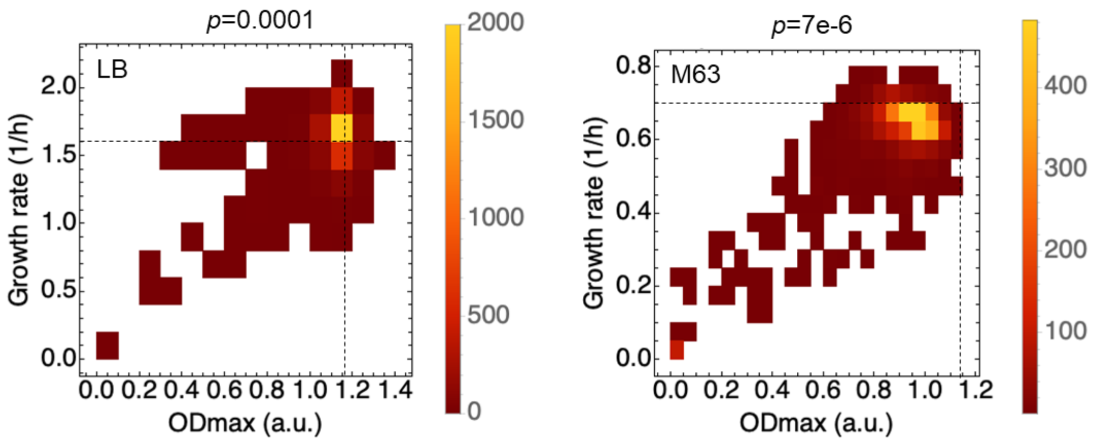

Figure S14 Density histograms of the growth profiles of the Keio collection strains used in the present study. The left and right panels present the growth profiles (N=3909) in LB and M63 media, respectively. The colour gradation from red to yellow indicates the number of strains from small to large. The statistical significance of the correlation is indicated. The growth rate and the maximal OD_{600} of the wild-type strain are indicated by the broken lines.

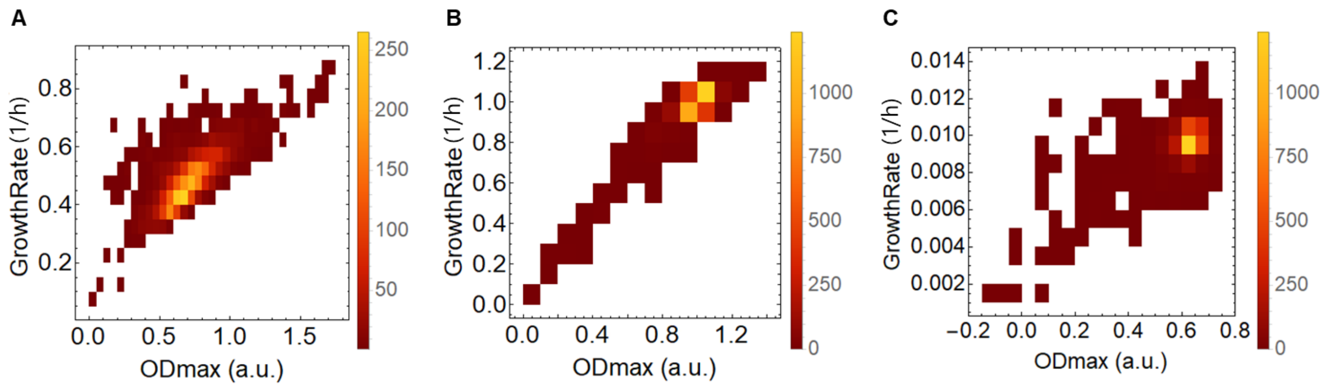

Figure S15 Density histograms of the growth profiles of the Keio collection strains in other reports. The data sets were obtained from previous studies reporting the growth profiles of the Keio collection strains. The growth assays were performed under the following conditions: **A.** M9 medium, 384-well (N=3907); **B.** LB agar medium, colony formation (N=3350); and **C.** M9 medium, single cell (N=3301). The relative values of the growth rates and the maximal population densities are shown. The colour gradation from red to yellow indicates the number of strains from small to large.

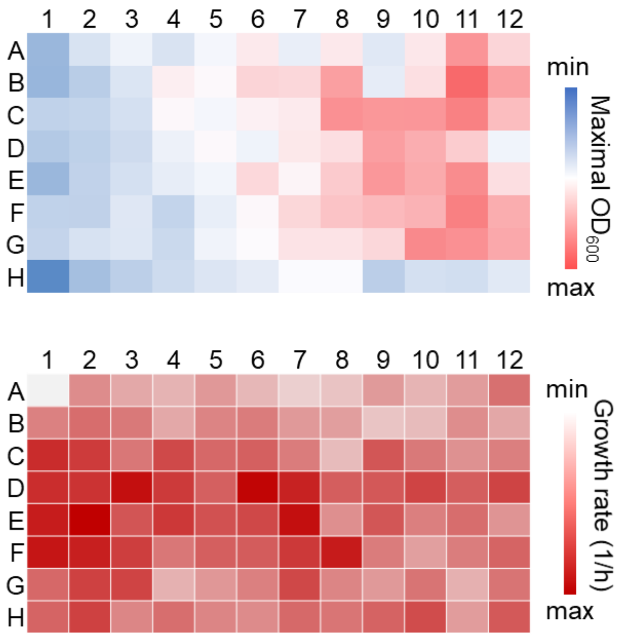

Figure S16 Experimental bias in the growth assay. The mean values of the maximal OD_{600} (upper panel) and the growth rates (bottom panel) of the knockout strains assayed in the same well are shown in a 96-well microplate format. Each well contains more than 50 genotypes and hundreds of growth assays. The colour gradation indicates the mean values of the growth rates or the maximal OD_{600} assayed in the individual wells.

Table S1 Genes with a very negative contribution to growth fitness. A total of 14 and 10 genes were determined to have a very negative contribution to growth in M63 and LB media, respectively. JWID, Genes, Growth, GC, ES and Function represent the ID number of the gene, the names of the missing genes, the growth rates of the knockout strains, the assigned gene category, the indicator of essentiality as reported by the constructors and the molecular function of the gene product, respectively.

Media	JWID	Genes	Growth/h	GC	ES	function
M63	JW0325	prpD	0.745	e	-4	2-methylcitrate dehydratase
	JW4286	uxuB	0.748	e	-4	D-mannonate oxidoreductase, NAD-binding
	JW1622	rsxD	0.75	pe	-3	predicted inner membrane oxidoreductase
	JW5458	ygeK	0.75	pr	-2.5	predicted DNA-binding transcriptional regulator
	JW3651	yidG	0.753	pm	-2	predicted inner membrane protein
	JW3148	greA	0.753	f	-4	transcription elongation factor
	JW3474	slp	0.753	lp	-2	outer membrane lipoprotein
	JW2659	mprA	0.753	r	-4	DNA-binding transcriptional regulator
	JW1527	ydeE	0.755	pt	-3	predicted transporter
	JW5741	rnr	0.765	e	-4	exoribonuclease R, RNase R
	JW3228	dusB	0.765	e	-3	tRNA-dihydrouridine synthase B
	JW3561	aldB	0.769	e	-3.5	aldehyde dehydrogenase B
	JW3499	bcsZ	0.775	e	-3	endo-1,4-D-glucanase
	JW5397	hda	0.775	cp	0	ATPase regulatory factor involved in DnaA inactivation
LB	JW0229	frsA	1.998	r	-3	hydrolase, binds to enzyme IIA(Glc)
	JW0858	ybjE	2.138	pt	-4	predicted transporter
	JW1032	ymdB	2.079	o	-3	conserved protein
	JW1446	yncD	2.009	pt	-3	predicted iron outer membrane transporter
	JW1631	ydhA	2.083	lp	-3.5	predicted lipoprotein
	JW1940	yodC	1.985	o	-2.5	predicted protein
	JW2044	wcaA	2.04	pe	-4	predicted glycosyl transferase
	JW3454	yhiI	2.126	pt	-4	predicted HlyD family secretion protein

JW3869	frvX	1.992	pe	-3.5	predicted endo-1,4-beta-glucanase
JW5679	rhsB	1.99	h	-3	rhsB element core protein RshB

Table S2 Common genes between DEGs and DGGs. A total of 29 genes that overlapped between the DEGs and DGGs are summarized. JWID, Genes, GC and Function represent the ID number of the gene, the names of the genes, the assigned gene category and the molecular function of the gene product, respectively. DEGs LB/M63 and DGGs LB/M63 indicate the comparison of the gene expression (DEGs) and the growth rates (DGGs) between cells grown in LB and M63 media, respectively.

Genes	GC	DEGs LB/M63	DFGs LB/M63	JWID	function
argA	e	low	high	JW2786	fused acetylglutamate kinase homolog (inactive) -!- amino acid N-acetyltransferase
argB	e	low	high	JW5553	acetylglutamate kinase
argC	e	low	high	JW3930	N-acetyl-gamma-glutamylphosphate reductase, NAD(P)-binding
argG	e	low	high	JW3140	argininosuccinate synthetase
argH	e	low	high	JW3932	argininosuccinate lyase
bioB	e	low	high	JW0758	biotin synthase
bioC	pe	low	high	JW0760	predicted methltransferase, enzyme of biotin synthesis
bioD	e	low	high	JW0761	dethiobiotin synthetase
cysC	e	low	high	JW2720	adenosine 5'-phosphosulfate kinase
cysD	e	low	high	JW2722	sulfate adenylyltransferase, subunit 2
cysH	e	low	high	JW2732	3'-phosphoadenosine 5'-phosphosulfate reductase
cysI	e	low	high	JW2733	sulfite reductase, beta subunit, NAD(P)-binding
cysN	e	low	high	JW2721	sulfate adenylyltransferase, subunit 1
cysW	t	low	high	JW2416	sulfate/thiosulfate transporter subunit -!- membrane component of ABC superfamily
hisC	e	low	high	JW2003	histidinol-phosphate aminotransferase
ilvC	e	low	high	JW3747	ketol-acid reductoisomerase, NAD(P)-binding
leuA	e	low	high	JW0073	2-isopropylmalate synthase
leuB	e	low	high	JW5807	3-isopropylmalate dehydrogenase
leuC	e	low	high	JW0071	3-isopropylmalate isomerase subunit, dehydratase component
leuD	e	low	high	JW0070	3-isopropylmalate isomerase subunit
leuL	l	low	high	JW0074	leu operon leader peptide

metA	e	low	high	JW3973	homoserine transsuccinylase
metE	e	low	high	JW3805	5-methyltetrahydropteroyltriglutamate-homocysteine S-methyltransferase
metF	e	low	high	JW3913	5,10-methylenetetrahydrofolate reductase
serA	e	low	high	JW2880	D-3-phosphoglycerate dehydrogenase
trpA	e	low	high	JW1252	tryptophan synthase, alpha subunit
trpC	e	low	high	JW1254	fused indole-3-glycerolphosphate synthetase - N-(5-phosphoribosyl)anthranilate isomerase
trpD	e	low	high	JW1255	fused glutamine amidotransferase (component II) of anthranilate synthase - anthranilate phosphoribosyl transferase
trpE	e	low	high	JW1256	component I of anthranilate synthase
